# Graphitic carbon nitride (g-C_3_N_4_)-based photocatalytic materials for hydrogen evolution

**DOI:** 10.3389/fchem.2022.1048504

**Published:** 2022-10-25

**Authors:** Rui-Han Gao, Qingmei Ge, Nan Jiang, Hang Cong, Mao Liu, Yun-Qian Zhang

**Affiliations:** ^1^ Enterprise Technology Center of Guizhou Province, Guizhou University, Guiyang, China; ^2^ Key Laboratory of Macrocyclic and Supramolecular Chemistry of Guizhou Province, Guizhou University, Guiyang, China

**Keywords:** g-C3N4, photocatalysis, hydrogen evolution, energy materials, semiconductor

## Abstract

The semiconductors, such as TiO_2_, CdS, ZnO, BiVO_4_, graphene, produce good applications in photocatalytic water splitting for hydrogen production, and great progress have been made in the synthesis and modification of the materials. As a two-dimensional layered structure material, graphitic carbon nitride (g-C_3_N_4_), with the unique properties of high thermostability and chemical inertness, excellent semiconductive ability, affords good potential in photocatalytic hydrogen evolution. However, the related low efficiency of g-C_3_N_4_ with fast recombination rate of photogenerated charge carriers, limited visible-light absorption, and low surface area of prepared bulk g-C_3_N_4_, has called out the challenge issues to synthesize and modify novel g-C_3_N_4_-block photocatalyst. In this review, we have summarized several strategies to improve the photocatalytic performance of pristine g-C_3_N_4_ such as pH, morphology control, doping with metal or non-metal elements, metal deposition, constructing a heterojunction or homojunction, dye-sensitization, and so forth. The performances for photocatalytic hydrogen evolution and possible development of g-C_3_N_4_ materials are shared with the researchers interested in the relevant fields hereinto.

## 1 Introduction

With the development and progress of human society, environmental pollution and energy shortage have become two major problems that plague human beings. Hydrogen is considered as one of the best candidates for storing solar energy meeting the growing clean energy demand (Chen et al., 2016; Shen et al., 2016; Wang et al., 2016; Wu et al., 2017; Wang et al., 2018; Liu et al., 2019). Since Fujishima and Honda discovered the hydrogen evolution reaction activates by TiO_2_ under irradiation in 1972, photocatalytic water splitting is one of the promising means for hydrogen production (Fujishima and Honda., 1972). Without relying on fossil reserves, the photocatalytic hydrogen evolution from water with highly efficient utilization of solar irradiation is a desirable exploration for the solution of the energy issues (Maeda et al., 2006; Qi et al., 2022). Although great process in photocatalysts of water splitting have been made for H_2_ evolution under visible light, there are still challenging and concerns with semiconductors to promise hydrogen energy development methods (Zhong et al., 2015; Wang et al., 2016; Zhang et al., 2018; Qi et al., 2020).

Graphitic carbon nitride (g-C_3_N_4_) is considered as an ideal 2D material with the conjugated skeleton for photocatalytic water splitting with the activity of photoelectronic chemistry and high stability in the photochemical reaction (Ong et al., 2016). Compounds in rich carbon and nitrogen elements such as melamine, urea, cyanamide, dicyandiamide, cyanuric acid, etc. are usually subjected as the precursors. Graphitic carbon nitride materials were synthesized by methods including electro-chemical deposition, thermal shrinkage polymerization, solid phase synthesis, gas phase synthesis, solvothermal synthesis and electrochemical deposition (Thomas et al., 2008). Under light irradiation, electron-hole pairs were generated on the surface of g-C_3_N_4_ photocatalyst to provide the reaction sites. The water molecules adsorbed on the surface of g-C_3_N_4_ undergo the photocatalytic reduction for H_2_ evolution and oxidation for O_2_ release, respectively, with the efficacious charge carriers by the reactions (1–3):

Oxidation:

$$H_{2}O+2h^{+}\to 2H^{+}+1/2O_{2}$$ (1)

Reduction:

$$2H^{+}+2e\to H_{2}$$ (2)

Overall reaction:

$$H_{2}O\to H_{2}+1/2O_{2}$$ (3)

The first case of g-C_3_N_4_ as a polymeric photocatalyst for water splitting to produce H_2_ under visible-light irradiation was reported by Wang et al. (Wang et al., 2009) Figure 1 schematically described the photogeneration of H_2_ and O_2_ in water splitting reaction with the pristine g-C_3_N_4_. The obtained bulk form of g-C_3_N_4_ exhibited some drawbacks including limited visible light utilization efficiency, fast recombination rate of photogenerated electron-hole pairs, and low specific surface areas (<10 m^2^g^−1^), which still limited the photocatalytic performance of on its practical applications (Reza et al., 2015; Fu et al., 2018), and modification of g-C_3_N_4_ has been recognized to be the effective way to improve the photocatalytic performance of pristine g-C_3_N_4_.

**FIGURE 1:**
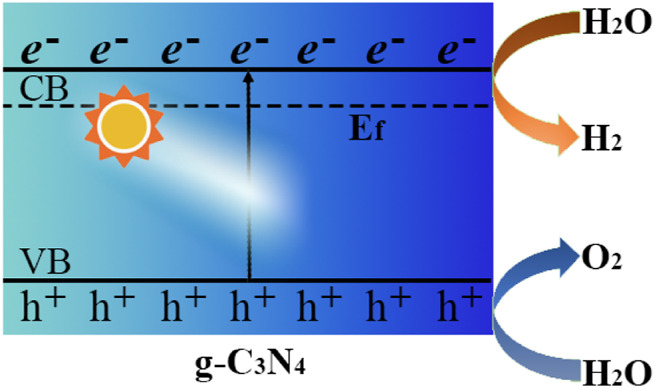
Schematic of generation of H_2_ and O_2_ from water with the catalysis of pristine g-C_3_N_4_ under light irradiation.

## 2 Modification of graphitic carbon nitride materials

Recently, the application of g-C_3_N_4_ with improved photocatalytic performance by developed several strategies, involving adjusting pH value, morphology control, doping by heteroatoms or metals, participation of co-catalyst, dye-sensitization, and construction of heterojunction. The hydrogen evolution performance of the modified g-C_3_N_4_-based materials are summarized in Table 1 to provide the development of the co-catalysts in the photolysis system.

**TABLE 1:** Hydrogen evolution performance of the modified g-C_3_N_4_-based materials.

Methods	Co-catalysts	Hydrogen evolution rate (μmol h^−1^g^−1^)	Ref.
0D	Quantum dots	2,199.2	Wang et al. (2014)
1D	Nanotubes	11,850	Mo et al. (2018)
2D	Nanosheets	3,140	Zhao et al. (2018)
3D	Nanovesicles	10,300	Sun et al. (2022a)
Non-metal doping	P dopant	1,596	Ran et al. (2015)
Metal doping	Co dopant	560	Chen et al. (2017)
Metal deposition	Pt co-catalyst	947.64	Zhu et al. (2019)
Dye sensitization	Protoporphyrin	1,153.8	Liu et al. (2020)
Heterogeneous	CeO_2_	1,240.9	Zhao et al. (2021)
Homojunction	High-crystalline g-C_3_N_4_	5,534	Sun et al. (2022b)

### 2.1 pH

**FIGURE 2:**
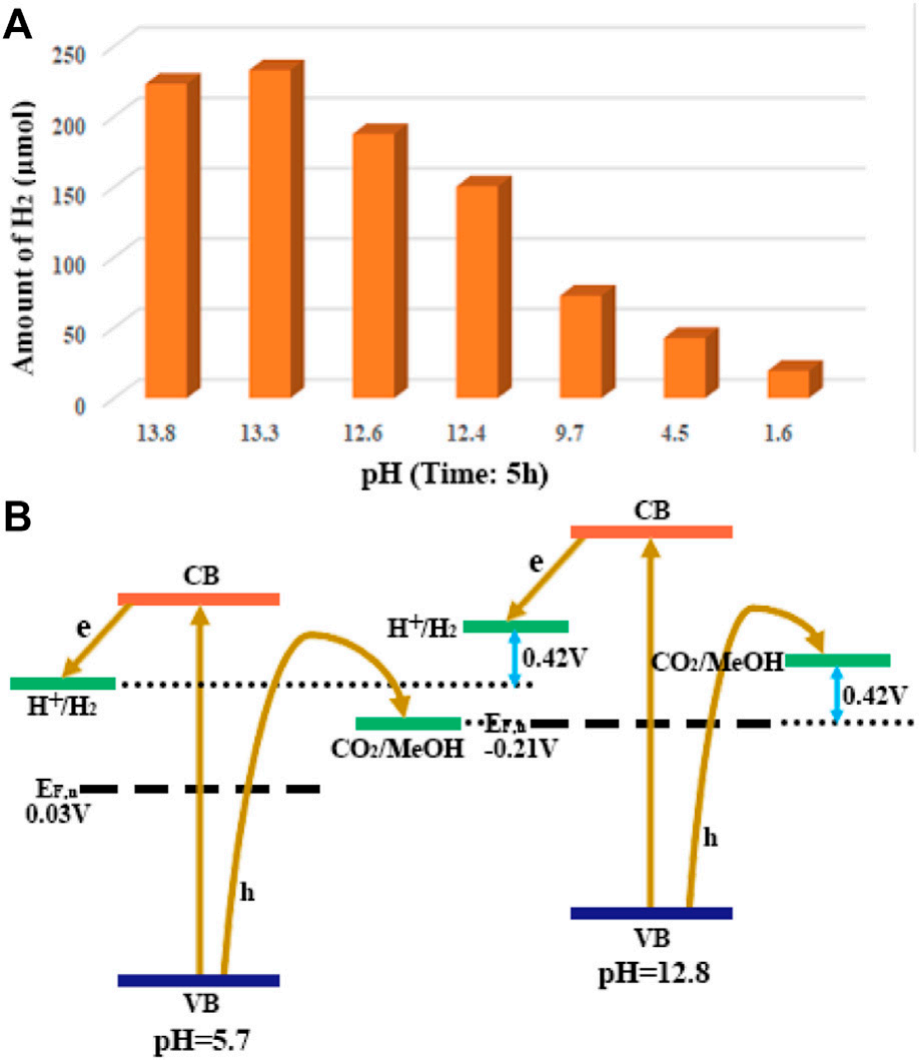
**(A)** the relationship of pH values to the efficiencies of H_2_ evolution with g-C_3_N_4_ photolysis; **(B)** energy diagrams of g-C_3_N_4_ in solution at pH 5.7 and 12.8.

The pH value of solution was an important factor affecting the activity of g-C_3_N_4_, that is, Zeta potential values suggested the surface charge of g-C_3_N_4_ could be changes at different pH value for the diversity of functional groups on the surface (Wang et al., 2016). Wu et al. demonstrated that the alkaline environment was beneficial to the photocatalytic hydrogen evolution efficiency of g-C_3_N_4_ material as shown in Figure 2A (Wu et al., 2014). The experimental results show that pH and methanol have certain effects on the photocurrent amplification on g-C_3_N_4_ films. In the presence of methanol, the photoelectronic efficiency was improved to provide an increased photocurrent from 0.6 to 1.2 μA cm^−2^, which was further enhanced to offer a 4.2 μA cm^−2^ current upon adding base to bring the pH to 12.8. The results implied the transfer of photogenerated holes into solution was enhanced by the addition of methanol and alkali, which could root in the additive-induced decrease of the energy gap of the flat band and band-edge of g-C_3_N_4_ as description in Figure 2B, that is, methanol oxidation occurred in alkaline solution, but restrained in acidic condition with the amine-terminated g-C_3_N_4_ surface.

### 2.2 Morphology control

**FIGURE 3:**
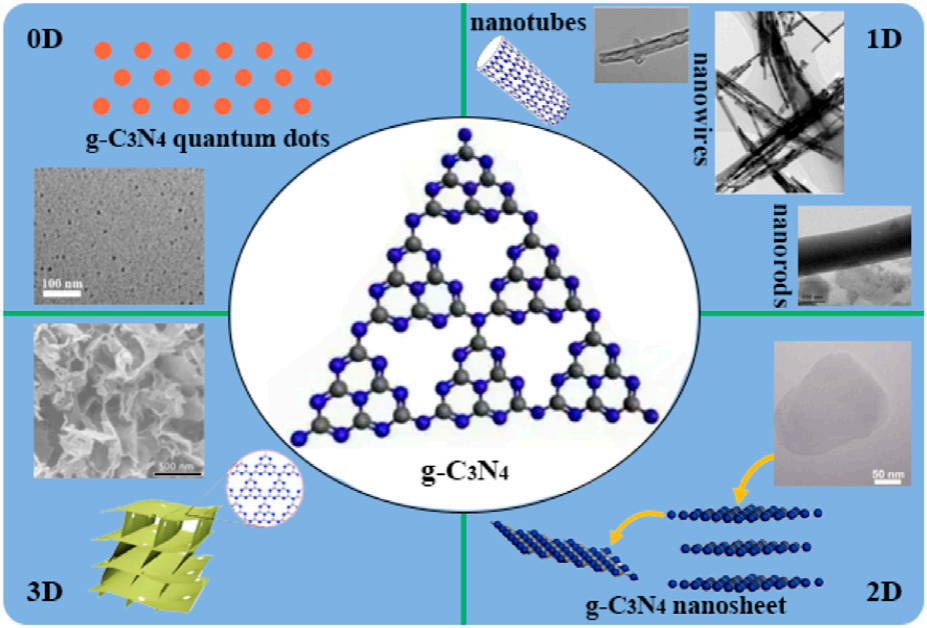
Schematic illustration of 0D, 1D, 2D and 3D g-C_3_N_4_.

The activity of g-C_3_N_4_ for H_2_ production *via* water splitting under visible-light irradiation could be determined by morphology of the material surface (Niu et al., 2012; Zhang et al., 2012; Han et al., 2015). The targets of controllable morphologies in preparation of well-defined g-C_3_N_4_ nanostructures to get larger specific surface area and more abundant reactive sites, reduced the recombination rate of photogenerated charge carriers. There were different nanostructures of g-C_3_N_4_ have been described in pioneering reports involving zero-dimensional (Wang et al., 2014) (0D), one-dimensional (Bai et al., 2013; Zhang et al., 2013; Wang et al., 2015; Mo et al., 2018; Bashir et al., 2019; Zhang et al., 2020) (1D), two-dimensional (Li et al., 2015; Zhao et al., 2018; Qi et al., 2019; Shi et al., 2022) (2D), three-dimensional (Li et al., 2016; Di et al., 2018; Chen et al., 2019) (3D) as shown in Figure 3, which built an ideal platform for collectively advanced photoredox processes for the enormous advantages in terms of physical and chemical characterization in following details.

The photocatalytic performance of 0D nanostructured materials are dependence on the natures including quantum size effect, small size effect, surface effect, macroscopic quantum effect and so on. Prof. Yu and co-workers prepared graphitic carbon nitride quantum dot structures directly from g-C_3_N_4_ with a thermochemical etching process, which produced unique upconversion properties and higher hydrogen production efficiency than original g-C_3_N_4_ in 2.87 times (Wang et al., 2014).

There was more explosion of active sites on the surface of 1D g-C_3_N_4_, which was reported as nanotubes (Mo et al., 2018; Guo et al., 2021), nanowires (Zhang et al., 2013; Wang et al., 2015), nanorods (Bai et al., 2013; Bashir et al., 2019) and so on, and efficient transfer of photogenerated electrons could be realized along one-dimensional paths with enhancement of visible light absorption and fast short-distance electron transport. Mo et al. developed g-C_3_N_4_ nanotubes with large number of nitrogen defects by a green-, acid- and base-free synthesis method, and the hydrogen production of 118.5 μmol h^−1^ was far superior to pristine g-C_3_N_4_ (Mo et al., 2018).

Compared with 1D structures, 2D photocatalysts have greater potential because of their larger specific surface area and thinner thickness, exposing more active sites and shortening the transport path of photogenerated carriers. Prof. Zhu’s group successfully fabricated g-C_3_N_4_ nanosheets with a single atomic layer structure of only 0.4 nm thickness, with a simple chemical exfoliation method. The single-atom-layer nanosheets offered better separation and transfer rates of photogenerated carriers, and exhibited higher performance than bulk g-C_3_N_4_ in photocatalytic splitting of water for hydrogen production and photocurrent generation. Chen’s group proposed that the precursors assembled into nanorods at low power level, while grew into nanoplates at high power level, which implied that the morphology of g-C_3_N_4_ was dependance upon on a kinetically driven process (Li et al., 2015). Zhao et al. treated supramolecular precursors under the action of glycerol and ethanol to obtain porous few-layer g-C_3_N_4_ (Zhao et al., 2018). The hydrogen evolution rate of thin-layer g-C_3_N_4_ was evaluated to be 159.8 μmol h^−1^, as the results of its large specific surface area, more active sites, and the abundant nitrogen vacancies in the framework, to accelerate the transfer of photogenerated electrons.

Compared with 2D g-C_3_N_4_ nanosheets, the porous 3D g-C_3_N_4_ material can provide a larger specific surface area. It also maximizes the use of incident photons through multiple reflections within the interconnected open frame (Di et al., 2018). In addition, the porous 3D g-C_3_N_4_ material acted as a support to prevent the agglomeration of ultrathin nanosheets and provided a pathway for electron transfer, thereby greatly enhancing the photocatalytic activity (Li et al., 2016). Zhang et al. utilized a simple bottom-up supramolecular self-assembly route to assemble a porous 3D g-C_3_N_4_ with high crystallinity and applied it to photocatalytic water splitting (Chen et al., 2019). In 2022, Guo et al. reported a facile template-free self-assembly method to synthesize three-dimensional porous g-C_3_N_4_ nanovesicles for achieving efficient and durable photocatalytic generation of H_2_, and the large-size vesicles exhibited the high H_2_ production rate of 10.3 mmol h^−1^ g^−1^ (Sun et al., 2022). And 3D onion-ring-like g-C_3_N_4_ was made from silica microsphere as a hard-template, which affored excellent properties such as large specific surface area, strong optical absorption, high dispersion, for the efficient water splitting with 5-fold higher than that of pristine g-C_3_N_4_ (Cui et al., 2018; Shi et al., 2022).

### 2.3 Doping

Graphitic carbon nitride, as a conjugated polymeric material with a band gap of about 2.7 eV, has a relatively narrow response to visible-light. Numerous research results suggested that the optical properties and some other physical properties of g-C_3_N_4_ could be well regulated by doping foreign elements (Chen et al., 2017; Zeng et al., 2018; Fang et al., 2019; Sun H.,R. et al., 2022a). Therefore, the photocatalytic activity of pure g-C_3_N_4_ could be improved by hybridization with a small amounts of non-metals or metals into the framework.

#### 2.3.1 Non-Metal doping

Hybridization of non-metallic dopants such as B, S, O, P and I to realize the ingenious design of the electronic structure, was considered as an important method for the improvement of g-C_3_N_4_ performance (Qi et al., 2021). Non-metal doping refers to doping of some non-metal elements into the structural framework, which not only modified the electronic and textural properties of g-C_3_N_4_ photocatalyst, but also improved the separation efficiency of photogenerated charge carriers and finally boosted the photocatalytic activity. Fang and coworkers (Fang et al., 2019) reported P-doped g-C_3_N_4_ for photocatalytic water splitting, and 4-(diphenylphosphino)benzoic acid (4-DPPBA) was employed as the precursor of phosphorous. The combination of P-doping and thermal exfoliation was applied for the preparation of porous g-C_3_N_4_ with P hybridization, which afford excellent photocatalysis for hydrogen evolution high to 1,596 μmol h^−1^ g^−1^ under irradiation of visible light (Ran et al., 2015). As demonstrated by DFT and experimental studies, the empty intermediate bandgap state enhanced the photo sensitivity with P hybridization, and the mass transfer process and light trapping were improved on the macroporous structure. The intrinsic energy gap of g-C_3_N_4_ was decrease from 2.98 to 2.66 eV in the attendance of P dopant. On the other hand, Lin and co-workers discovered the B,F-doped g-C_3_N_4_ porous nanosheets were achieved by the self-polymerization of urea in the presence of ionic liquid [Bmim][BF_4_], which yielded photocatalytic hydrogen in 3.9 times higher than pristine g-C_3_N_4_ (Lin and Wang., 2014) (Zhang et al., 2014). successfully obtained iodine-doped carbon nitride (CN-I) with calcining dicyandiamide to significantly improve the hydrogen production performance (Zhang et al., 2014). The photocatalytic activity of iodine-doped g-C_3_N_4_ was occurred at the wavelength of 600 nm, while pristine g-C_3_N_4_ provided inactive catalysis at 500 nm. Guo et al. (Guo et al., 2016) prepared a phosphorus-doped hexagonal hollow tubular structure g-C_3_N_4_ by hydrothermal method and the special structure greatly increased the specific surface area of the catalyst, thereby increasing the number of active sites for hydrogen production. Carbon doping is also an important part of non-metal dopants. In 2021, Liu et al. reported the synthesis of C-doped g-C_3_N_4_ by one-step copolymerization using melamine and chitosan as the raw materials (Liu et al., 2021). The N atom in g-C_3_N_4_ matrix was replaced by C to form the delocalized big Π bonds. The prepared C-doped g-C_3_N_4_ exhibited an excellent photocatalytic H_2_ evolution activity of 1,224 mmol h^−1^ g^−1^, which was 4.5 times than the free g-C_3_N_4_.

#### 2.3.2 Metal doping

In addition to the doping of non-metallic elements, the g-C_3_N_4_ framework was also doped with metallic elements to modify the electronic energy band structure, thereby improving the visible-light absorption, and enhancing the migration and separation of photogenerated carriers in the g-C_3_N_4_ photocatalyst (Chen et al., 2016). reported the Co-doped g-C_3_N_4_ synthesized by one-step thermal polymerization of cobalt phthalocyanine (CoPc) and melamine as the precursors (Chen et al., 2017). Yue et al. used a simple chemical method to dope metallic Zn into g-C_3_N_4_ (Yue et al., 2011). When the content of Zn was 10%, the visible-light-generated hydrogen production activity was 10 times higher than the pure g-C_3_N_4_. The proposed mechanism implied that doping of Zn increased the light absorption, improved the separation efficiency of electron-hole pairs, and enabled more electrons for water splitting. However, it is still a challenge to obtain nanoparticles with uniform size, regular shape, and high stability with common precursors such as polymers, carbon supports, ionic liquids, surfactants and microemulsions. In the recent report of our group, the coordination complex of cucurbit [6]uril and Co^2+^ was developed as the precursor, to produce cobalt nanoparticles with themolysis, for photocatalytic electrolysis of water by deposition on the surface of the g-C_3_N_4_ film. (Dai et al., 2022). The formed semiconductor-metal interface provided more reaction sites and electron transport channels for effective charge carriers to capture photons and excite electrons, thereby, promoting the photoelectrocatalytic reaction process. The discovery provided a new strategy for exploring macrocyclic/g-C_3_N_4_ materials with excellent photocatalytic activities.

### 2.4 Metal deposition (co-catalyst)

Various studies suggested that metal deposition on pure g-C_3_N_4_ was also one of the promising methods to enhance the photocatalytic activity. In theory, when metal nanoparticles are in contact with g-C_3_N_4_, a Schottky junction is formed at the interface of metal and g-C_3_N_4_ semiconductor due to the different work function, which changes the electron distribution on the semiconductor surface (Naseri et al., 2017; Caux et al., 2019; Qi et al., 2020; Zhao et al., 2021). The main function of metal is to accept the photogenerated electrons from the CB of g-C_3_N_4_ during the photocatalytic H_2_ production process. Various metals such as Pt (Ou et al., 2017; Zhu et al., 2019), Au (Samanta et al., 2014; Caux et al., 2019), Pd (Xiao et al., 2019), Ag (Nagajyothi et al., 2017; Deeksha et al., 2021) and Ni (Indra et al., 2016; Kong et al., 2016) were employed as co-catalyst for the efficient sensitization for photocatalysis with the surface plasmon resonance (SPR) effect, which improved the light absorption capacity of the catalyst. With an *in situ* photoreduction, Pt/g-C_3_N_4_ was subjected to be a visible light photocatalyst by Wang’s group, and the results indicated that the photocatalytic hydrogen evolution capability was gradually enhanced as the size decrease of the Pt co-catalyst (Zhu et al., 2019). The participation of Pt provided more active sites on the surface for reduction, which was favorable for accepting electrons from CB of g-C_3_N_4_, due to the formation of Schottky junctions at the interface of Pt and g-C_3_N_4_. The PL spectra and UV-vis/DRS spectra of g-C_3_N_4_ and Pt_x_-CN with different Pt content, demonstrated that Pt loading greatly improved charge separation and transfer in g-C_3_N_4_ photocatalysts, thereby reduced charge recombination, and enhanced photocatalytic activity, as well as provided the maximum utilization efficiency photocatalytic performance for H_2_ production. The Pt_0.1_-CN (with 0.1wt% Pt loading amount) sample displayed the highest photocatalytic activity with H_2_ evolution of 473.82 μmol mg^−1^ under visible-light irradiation.

**FIGURE 4:**
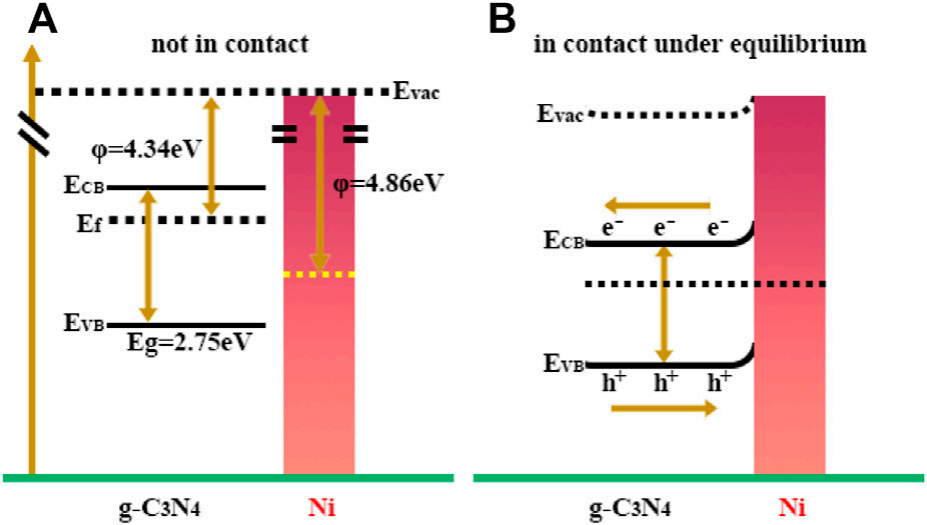
Schematic graph shows **(A)** the energy level diagram of g-C_3_N_4_ and Ni; **(B)** the interfacial electron transfer between g-C_3_N_4_ and Ni under irradiation.

Furthermore, Bi et al. reported a Ni cocatalyst for the enhancement of photocatalytic performance of g-C_3_N_4_ (Bi et al., 2015). A higher separation efficiency of photogenerated charge carriers was obtained as a result of a deeper band bending of g-C_3_N_4_ contacting with Ni, which contributed to enhanced photocatalytic H_2_ production performance. In addition, the heterojunction formed between the Ni nanoparticles and g-C_3_N_4_ acted as an electron collector, and impeded the recombination rate of photogenerated electron and holes as illumination in Figure 4. Ni/g-C_3_N_4_ catalyst exhibited high photocatalytic H_2_ evolution rate (8.314 μmol h^−1^) compared with pristine g-C_3_N_4_, in which rapid recombination between conduction band (CB) and valence band (VB) holes and the quick reversible reaction occurred.

### 2.5 Dye sensitization

To overcome the g-C_3_N_4_ absorption edge of a band gap of 2.7 eV, organic dyes were employed as a driver to improve the visible-light photoactivity (Bard and Fax., 1995; Kudo and Miseki., 2009; Kim et al., 2015), which were considered to dramatically extend the visible-light region of the band-gap of semiconductor (Zhuang et al., 2019). However, the researches about H_2_ production based on dye-sensitized carbon nitride were still insufficient, only few organic dyes such as metal-porphyrins (Yu et al., 2014; Chen et al., 2015; Zhang et al., 2015; Zhuang et al., 2019; Liu et al., 2020), poly (3-hexylthiophene) (Zhang et al., 2015), eosin Y (EY) (Min and Liu., 2012; Wang et al., 2018; Qi et al., 2019; Xu et al., 2019; Nagaraja et al., 2020; Zhao et al., 2021) and erythrosin B (ErB) (Wang et al., 2013; Zhang et al., 2017; Zhang et al., 2017) have been successfully applied to enhance the photocatalytic activity with improvement of the utilization efficiency of visible-light. In the process of H_2_ generation, the organic dyes were damaged in oxidation reactions, and its stabilization could be realized with a porous support, which accelerates the transfer of electrons from the excited dye molecule to the active site in definition of a cocatalyst, in general use of noble metals (especially Pt). The hybridization of Ag with g-C_3_N_4_ was applied for hydrogen evolution, and the photocatalysis was improved with the dye-sensitization under visible-light irradiation (Schwinghanmmer et al., 2013). Min et al. reported that g-C_3_N_4_ with modification of Eosin Y performed the light-drove H_2_ generation at about 600 nm, while the reaction occurred at less than 460 nm on the pristine g-C_3_N_4_ surface (Min and Liu., 2012).

### 2.6 Heterogeneous structure

The photocatalytic efficiency and application of pristine g-C_3_N_4_ were limited for high recombination rate of photogenerated charge carriers and narrow range of visible light response in a solar spectrum. Recently, g-C_3_N_4_-based heterojunctions were developed by enhancement of carrier separation efficiency and demonstrated excellent photocatalytic performance. Semiconductors were induced to form heterojunctions with g-C_3_N_4_ including carbon materials (graphene (Xiang et al., 2011), carbon nanotubes (Ge and Han., 2012), fullerenes (Chai et al., 2014)), metal oxides (TiO_2_(Chen and Liu, 2016), SnO_2_(Zada et al., 2019), ZnO(Sun et al., 2012), NiFe_2_O_4_(Liu et al., 2022), Fe_2_O_3_(Theerthagiri et al., 2014)), metal sulfides (CdS(Chen et al., 2016), ZnS(Shi et al., 2014), MoS_2_(Li et al., 2014)), bismuth-based compounds (BiPO_4_(Zou et al., 2015), BiVO_4_(Li et al., 2014), Bi_2_WO_6_(Li et al., 2018)), silver-based compounds (Ag_2_O(Liang et al., 2019), Ag_3_PO_4_(Liu et al., 2016), Ag_3_VO_4_(Zhu et al., 2015)), multi-element rare Earth oxides (Zn_2_GeO_4_(Sun et al., 2014), SrTiO_3_(Xu et al., 2011)), etc. The principle was executed in design of the heterojunction, that is, the recombination of g-C_3_N_4_ and the band-matched semiconductor promoted the transfer of charge carriers and suppressed the recombination of charges.

**FIGURE 5:**
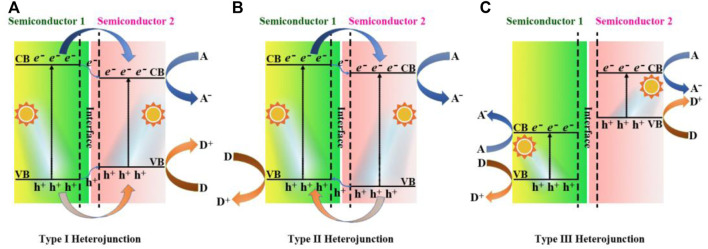
Schematic representation band structure of different heterojunctions: **(A)** Type Ⅰ heterojunction; **(B)** Type Ⅱ heterojunction; **(C)** Type Ⅲ heterojunction. A and D represent electron acceptor and electron donor, respectively.

Based on different photogenerated carrier transfer mechanisms, the heterojunctions were formed when g-C_3_N_4_ coupled with other materials (Reza et al., 2015; Patnaik et al., 2016; Fu et al., 2018). In the heterojunction structures, Type-Ⅰ constructure refers to that the position of CB of semiconductor-1 is higher than that of semiconductor-2, while the VB position of semiconductor-1 is lower than that of semiconductor-2, as shown in Figure 5A. Under the excitation of visible light, electrons and holes of the Type-Ⅰ heterojunction photocatalyst are more inclined to migrate to the semiconductor-2 with a smaller band gap and undergo a redox reaction, and the separation efficiency of carriers is not significantly improved, resulting in the low rate of photocatalytic redox reaction. In the Type-Ⅱ structure, the positions of both CB and VB of semiconductor-2 are lower than those of semiconductor-1, and therefore the photogenerated electrons and holes transferr into different sides of the heterostructure, as shown in Figure 5B. The carrier transport mode of the Type-Ⅱ heterojunction greatly improve the photocatalytic activity of the composite photocatalyst. In 2021, Roy’s team reported the TiO_2_/ultrathin g-C_3_N_4_ (U-g-CN) heterostructure photocatalyst using a unique *in situ* thermal exfoliation process, and the presence of U-g-CN produced a redshift (∼0.13eV) in the absorption edge of heterostructures compared to that of bare TiO_2_, which extended the light absorption capability. Combined with the morphological characteristics of g-C_3_N_4_, Chen et al. prepared a novel 3D hierarchical hollow tubular g-C_3_N_4_/ZnIn_2_S_4_ nanosheets as the type-Ⅱ heterojunction photocatalyst (Chen et al., 2022). The optimum photocatalyst offered the H_2_ evolution rate up to 20,738 μmol h^−1^ g^−1^. In the case of the Type-Ⅲ heterojunction (Figure 5C), there are no any energy band intersection of semiconductor-1 and semiconductor-2, resulting in the inability of transport of photogenerated carriers between the semiconductors to greatly improve the photocatalytic efficiency.

**FIGURE 6:**
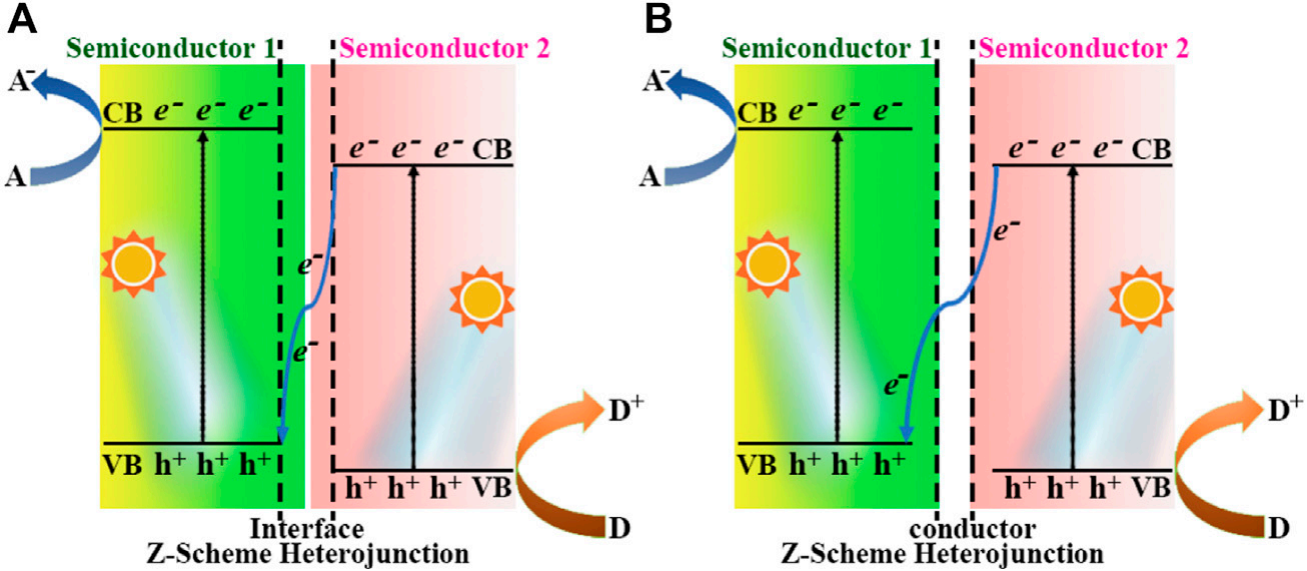
Schematic energy band diagram of two different types of Z-scheme heterojunction: **(A)** binary Z-scheme heterojunction, **(B)** ternary Z-scheme heterojunction. A and D denote electron acceptor and electron donor, respectively.

Thus, a suitable semiconductor heterojunction is able to both enhance the ability to capture sunlight and significantly accelerate the separation and migration of photogenerated electron-hole pairs as description in Type II structure, but it is still insufficient in terms of photocatalytic oxidation ability. The Z-scheme heterojunction (Xu et al., 2022) was explored to overcome this disadvantage to a certain extent, which was mainly divided into binary and ternary structures, as shown in the Figure 6 (Maeda, 2013). The CB and VB potentials of semiconductor-2 were more positive than those of semiconductor-1 in binary Z-scheme (Figure 6A), thereby enhancing the reduction and oxidation capacity of e^−^ and h^+^. Zhao et al. prepared the CeO_2_/g-C_3_N_4_ heterojunction photocatalysts, through a one-step *in situ* pyrolysis formation of 3D hollow CeO_2_ mesoporous nanospheres and 2D g-C_3_N_4_ nanosheets. The hydrogen evolution from water splitting experiment of the CeO_2_/g-C_3_N_4_-6 gave a maximum yield of 1,240.9 μmol g^−1^ h^−1^, which was about 5.2 times higher than that of CeO_2_ (Zhao et al., 2021). Figure 6B pictured out a conductor was employed as a charge bridge between the VB of semiconductor-1 and the CB of semiconductor-2 in the ternary Z-scheme heterojunction, which was played by metal particles, such as Cu, Au, Ag, etc. Hieu et al. synthesized the TiO_2_/Ti_3_C_2_/g-C_3_N_4_ (TTC) photocatalyst from g-C_3_N_4_ and Ti_3_C_2_ MXene *via* a calicination technique, and a high H_2_ production of 2,592 μmol g^−1^ was achieved (Hieu et al., 2021).

### 2.7 Homojunction structure

The g-C_3_N_4_ homojunctions are also recognized as the efficient photocatalysts. However, the type II structures and *Z*-schemes in the pioneering reports require deep optimization of the electron transport path in g-C_3_N_4_ homojunctions, since the redox potentials were depressed to inhibit the improvement of photocatalytic performance. The barrier could be overcome by the inspiration of *S*-scheme heterojunction proposed by Yu’s group (Xu et al., 2020), Guo et al. fabricated the *S*-scheme homojunctions with high-crystalline/amorphous g-C_3_N_4_ (HCCN/ACN) with solvothermal method, which was applied in photocatalytic H_2_ production with the evolution rates of 5.534 mmol h^−1^ g^−1^ in water and 3.147 mmol h^−1^ g^−1^ in seawater (Sun et al., 2022).

## 3 Conclusion

The excessive use and combustion of fossil fuels will inevitably bring some environmental problems. The value of hydrogen energy has been fully recognized, but its preparation technology still needs to be further explored. Photocatalytic technology is expected to realize sustainable energy production under the premise of making full use of solar energy, and has great potential in terms of energy and environment. The main factor limiting the photocatalytic activity of pristine g-C_3_N_4_ is its bulk structure, resulting in its small specific surface area and few active sites, which prolongs the transfer path of photogenerated electrons, thus accelerates the photogenerated charge carriers compound odds. The ability of photocatalytic hydrogen production performance of g-C_3_N_4_ could be improved by adjusting pH of the environment to induce the change of the surface charge of g-C_3_N_4_, controlling the morphology of g-C_3_N_4_ to increase active sites and shorten the transport path of carriers, and compositing co-catalysts or narrow-band semiconductors or dyes to enhance light absorption and reduce the recombination of photogenerated electrons and holes. So far, the strategies for exploration of stable hybridization structures to boost the photocatalytic efficiency could be the main concern in this filed, and more cases should be discovered to realize the dependence of the morphologies, structures, and species of dopants on the activities. This review is aimed at summarization of the recent progress of preparation and performance of g-C_3_N_4_-block photocatalysts to induce new ideas for the structural design with further improved efficiency by interdisciplinary researches across chemistry, physics, and material science.
