# Supplementary material for: Graphitic carbon nitride (g-C3N4)-based photocatalytic materials for hydrogen evolution
Source: Front Chem. 2022 Oct 25;10:1048504. doi: 10.3389/fchem.2022.1048504 (PMC9640947; doi:10.3389/fchem.2022.1048504)
Supplement: Supplementary file 1 [file DataSheet1.docx]

**Graphitic Carbon Nitride (g-C_3_N_4_)-based** **Photocatalytic Materials for Hydrogen Evolution**

Rui-Han Gao, Qingmei Ge, Nan Jiang, Hang Cong*, Mao Liu, Yun-Qian Zhang*


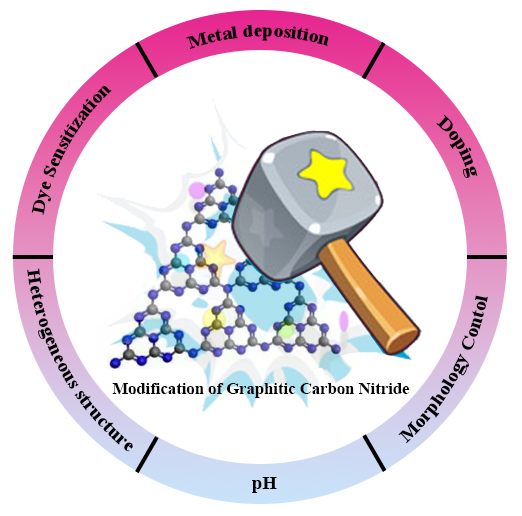


The improvement of photocatalytic reactivity of pristine g-C_3_N_4_ by adjustment of morphology, condition, and hybridization was summarized in this review, which were applied for photocatalytic hydrogen evolution reactions.
